# The Dialectics of Free Energy Minimization

**DOI:** 10.3389/fnsys.2019.00042

**Published:** 2019-09-10

**Authors:** Evert A. Boonstra, Heleen A. Slagter

**Affiliations:** Department of Experimental and Applied Psychology, Vrije Universiteit Amsterdam, Institute for Brain and Behavior Amsterdam (iBBA), Amsterdam, Netherlands

**Keywords:** Hegel, Friston, free energy minimization, dialectics, plasticity

## Abstract

Karl Friston’s free energy minimization has been received with great enthusiasm. With good reason: it not only makes the bold claim to a unifying theory of the brain, but it is presented as an *a priori* principle applicable to living systems in general. In this article, we set out to show how the breadth of scope of Friston’s framework converges with the dialectics of Georg Hegel. Through an appeal to the work of Catherine Malabou, we aim to demonstrate how Friston not only reinvigorates Hegelian dialectics from the perspective of neuroscience, but that the implicit alignment with Hegel necessitates a reading of free energy minimization from the perspective of Hegel’s speculative philosophy. It is this reading that moves beyond the discussion between cognitivism and enactivism surrounding Friston’s framework; beyond the question whether the organism is a secluded entity separated from its surroundings, or whether it is a dynamical system characterized by perpetual openness and mutual exchange. From a Hegelian perspective, it is the tension between both positions itself that is operative at the level of the organism; as a contradiction the organism sustains over the course of its life. Not only does the organism’s secluded existence depend on a perpetual relation with its surroundings, but the condition for there to be such a relation is the existence of a secluded entity. We intend to show how this contradiction—tension internalized—is at the center of Friston’s anticipatory organism; how it is this contradiction that grounds the perpetual process of free energy minimization.

## Introduction

This article moves in the interstices between neuroscience and contemporary readings of Georg Hegel’s philosophy (Catherine Malabou, Slavoj Žižek). It seeks to show how Karl Friston’s free energy minimization resuscitates Hegelian dialectics from the perspective of empirical science. Friston’s approach to neural functioning has revolutionized our understanding of the brain. From the perspective of free energy minimization, the brain is no longer conceived as an organ that merely incorporates influences from outside; instead, the brain is viewed as an anticipatory structure engaged in the continual process of its own maintenance and transformation (Friston, 2010). This approach to neural functioning not only sits well with what we know about the brain’s structure and functioning in terms of its anatomy and physiology, but the broader scope of Friston’s framework—as well as the discussions surrounding it—beg for a return to Hegel.

The connection between Hegelian philosophy and neuroscience is not new. We find an emblematic example of their convergence in Malabou’s book *What Should We Do with Our Brain?* from 2004, which revolves around the opposition between the Hegelian notion of plasticity on the one hand, and neuroplasticity on the other. Within neuroscience, we speak of neuroplasticity primarily in terms of the brain’s capacity to undergo change (e.g., Buonomano and Merzenich, 1998; Feldman, 2009). For example, in terms of synaptic connections strengthening or weakening dependent on their excitation. This account of plasticity emphasizes *passivity*: the degree to which the brain changes in response to influences it undergoes from outside; it is the brain’s capacity to *receive form*. Malabou (1996/2005) shows how in Hegel’s work, the notion of plasticity encompasses not only the designation of passively receiving form, but simultaneously implicates its obverse: the capacity to *produce* form. Said differently, plasticity harbors activity that often gets lost in its neural variant. And yet, advances in our scientific understanding of neuroplasticity also touch on the activity of plasticity, by moving beyond the passive association of synapses. For example, “homeostatic forms of neural plasticity regulate all the synapses on a neuron in unison in an orchestrated fashion” (Sweatt, 2016; p. 189). At the network level as well, sensory and motor systems have the capacity (to some extent) for cortical remapping in response to disease or stroke, through which some functioning may be recovered in the aftermath of such events (e.g., Wittenberg, 2010). For example, “[e]vidence in sensory systems was obtained in work on cross-modal plasticity in which the loss of input to one sensory modality resulted in cortical reorganization in other sensory systems” (Ostry and Gribble, 2016; p. 118). In both of these examples, we are dealing with a form of neuroplasticity implicating an interplay between different levels of organization, where more general levels of organization (neuron, network) may subsume the behavior of more specific processes (synapses, brain areas). Once a process operates at a more general level of organization, it may no longer make sense to insist on reducing its functioning to more specific levels (Bello-Morales and Delgado-García, 2015). And yet, reorganization can occur bidirectionally: a change in a specific process (e.g., one sensory modality) may give rise to reorganization in general. This kind of self-relating organizational process is relevant for the present article, because both in the case of Hegel and Friston, we are dealing with a bidirectional process of (active) organization and maintenance at the level of the brain or organism at large. The challenge at hand is to extend the brain’s organizational capacity to our most basic understanding of neural functioning, in order to move beyond a conception of the brain as a passive receiver of influences.

The challenge we face in neuroscience is to provide a formal description of neural functioning *from the standpoint of the brain*. This means that our explanation should take account of the limited access the brain has to its surroundings, as well as the (computational) constraints the brain is subject to. As we will see over the course of this article, Friston’s free energy minimization is a formal description of neural functioning that adheres to these constraints. The second advantage of Friston’s approach is that the framework is grounded in the minimal requirement for the existence and perpetuation of the brain itself: the imposition and maintenance of a boundary between the organism (including the brain) on the one hand, and the organism’s surroundings on the other. The result is that the brain starts to appear not as an organ of infinite malleability and accommodation; not as a flexible organ where “[a]ll that is solid, melts into air” (Marx and Engels, 1848/1888; p. 16). Instead, the brain appears as a self-organizing system that undergoes change as a way to *resist change*. Perhaps surprisingly, it is here where Hegel’s 19th century philosophy and Friston’s free energy minimization converge.

Indeed, perhaps it is the double meaning of plasticity—the capacity to receive form, and the capacity to produce form—which is at stake in free energy minimization. From this perspective, the brain is conceived as an anticipatory structure that actively models and anticipates its surroundings. An important implication is that both of plasticity’s capacities are implicated in the same circular process; namely, in the form of the reception of sensation, and in the production of sensation through action. Under free energy minimization, the brain is caught in a perpetual process of self-production, on the basis of which form is not only received from outside but is equally produced and maintained by the anticipatory structure itself. Friston’s framework allows us to see for the first time the significance of Malabou’s invocation of the Hegelian notion of plasticity *from the standpoint of neuroscience itself*. The elucidation of Friston’s free energy minimization against the backdrop of Malabou’s reading of Hegel will allow us to mobilize Malabou’s critique within a theoretical framework that has rapidly gained traction and stature within contemporary neuroscience.

There are two tenets from Malabou’s reading that need to be brought to light in order to bring out the convergence between Hegel and Friston. First, the primacy of *habit*, understood as a reduplication of nature, through which the organism starts to appear, already in Hegel, as an anticipatory structure. Second, the processual nature of the formation and maintenance of habit; a process that is at once plastic and dialectical. What makes the process of plasticity dialectical is that in it, contradictory moments coincide. The changing *formation* of a particular organism simultaneously implicates its *deformation*. Framed in terms of old and new, the “new defines itself in response to what is already established; at the same time, the established has to reconfigure itself in response to the new” (Fisher, 2009; p. 3). This contradictory process is what makes the organism (including the brain) dialectical.

But why appeal to Hegel in the first place? Why Hegelian dialectics? There are two reasons. The first reason is that already in his work, we find the designation of living organisms in terms of differentiating processes that presuppose the imposition of a boundary. In both the case of Hegel and Friston, it makes sense to speak of organic life only on account of the existence of a boundary which constitutes and sustains a separation between inside and outside. Contemporary readings of Hegel’s work suggest he was the first to fully anticipate contemporary conceptions of self-organization and self-production (*autopoiesis*). Due to the proximity in starting point and scope of both Hegel’s and Friston’s work, the first question we address is this: is free energy minimization a formalization of the dialectical process of plasticity, understood as the capacity both to receive and to produce form?

While Friston’s approach to neural functioning is incredibly attractive, our wager is that Friston himself (Allen and Friston, 2018), as well as the appropriators of his framework in terms of cognitivism (Hohwy, 2013, 2016; Wiese and Metzinger, 2017), and enactivism (Clark, 2013, 2015; Bruineberg et al., 2018), do not do justice to the unsolvable tension at the heart of neural functioning under free energy minimization: the brain’s anticipations are never “correct”; the brain necessarily and continuously sustains and attempts to solve the “errors” of its anticipations. There is no definite solution to this problem: the brain can only optimize its anticipations and thereby minimize its error. The result is that under free energy minimization, the brain sustains tension necessarily.

In addition to the brain’s unsolvable tension, the theoretical struggle surrounding Friston’s framework revolves around the tension between cognitivism and enactivism; between seclusion and openness (Hohwy, 2016; Allen and Friston, 2018). This tension revolves around the question whether the brain is secluded from its surroundings, or whether it is better characterized by openness to these surroundings, as part of a larger brain-body-environment system. We are not appealing to Hegel merely for historical reasons; the second reason to appeal to his work is that his philosophy provides us with a way to move beyond this discussion. How? From a Hegelian perspective, it is not enough simply to choose either cognitivism or enactivism; seclusion or openness. Or to insist on finding the right balance between the two. The appeal to Hegel allows us to see how Friston’s framework reintroduces the old Hegelian theme of a contradiction constitutive of life; an “Unbehagen in der Natur,” or a discontent in nature (Žižek, 2016; p. 350–351). The challenge is to show how the unsolvable minimization problem of Friston’s framework actualizes the tension between seclusion and openness. Said differently, we have to mobilize this tension at the level of the organism itself; to the point where it appears as a contradiction, the organism sustains over the course of its life. The second question central to this article is thus: can we enact the Hegelian shift from tension to contradiction with regards to free energy minimization?

The present article consists of three parts. First, after outlining the contradictory organism in Hegel, and the dialectical/plastic process of habitual anticipation as understood by Malabou (“Habitual Anticipation, Plasticity, Dialectics” section), we will move on to Friston’s conception of the anticipatory organism, in order to show how Friston’s free energy minimization converges with Malabou’s reading of Hegel (“States of Anticipation” section). Finally, we will try to move beyond both Friston and his cognitivist/enactivist appropriators, by enacting the shift from the organism’s unsolvable tension, to its constitutive contradiction (“From Tension to Contradiction” section).

## Habitual Anticipation, Plasticity, Dialectics

### Science and Teleology

The relationship between Hegelian dialectics and Friston’s free energy minimization touches on a larger discussion surrounding the connections between self-organization, self-production (*autopoiesis*), Friston’s framework, and predictive processing on the one hand, and Kant and Hegel on the other. The Kantian legacy at work in these notions, and in Friston’s approach to the brain, has received most attention within the philosophy of mind (Weber and Varela, 2002; Hohwy, 2013; Swanson, 2016; Wiese and Metzinger, 2017). While some attention has been devoted to the relationship between dialectics in general and predictive processing (Bolis and Schilbach, 2018), an investigation into the specifically Hegelian legacy at work in free energy minimization is long overdue. Especially since the connections between Hegelian dialectics and the notions of self-organization and *autopoiesis* are well-established (Žižek, 2006, 2012; Michelini, 2012; Marques, 2016; Michelini et al., 2018).

While Hegel can and should be criticized for relegating empirical science to something secondary (beneath philosophy), he by no means dismisses empirical science all together. On the contrary, his *Philosophy of Nature* is pervaded by the attempt to do justice to the science of his day: “[n]ot only must philosophy be in agreement with our empirical knowledge of Nature, but the *origin* and *formation* of the Philosophy of Nature presupposes and is conditioned by empirical physics” (Hegel, 1830/2004; p. 6, §246). However, Hegel lived long before the advent of non-equilibrium thermodynamics and cybernetics. The empirical physics in his time were Newtonian mechanics and the early days of thermodynamics. As such, Hegel followed Kant in problematizing the sufficiency of Newtonian mechanical laws for understanding living organisms (e.g., see Weber and Varela, 2002; Marques and Brito, 2014). In the words of Kant:

“An organized being is thus not a mere machine, for that has only a motive power, while the organized being possesses in itself a formative power, and indeed one that it communicates to the matter, which does not have it (it organizes the latter): thus it has a self-propagating formative power, which cannot be explained through the capacity for movement alone (that is, mechanism)” (Kant, 1790/2002, p. 246, §65).

A central issue surrounding the “self-propagating formative power” of organisms in contrast to Newtonian mechanics, is the problem of teleology, purpose, or “natural ends” [*Naturzweck*]. For Kant, a “thing exists as a natural end *if it is cause and effect of itself*” (Kant, 1790/2002, p. 243, §64). More precisely: “[a]n organized product of nature is that in which everything is an end and reciprocally a means” (Kant, 1790/2002, p. 247, §66). In other words, the question of purposiveness in nature pertains to the way organisms exert purpose in the conservation and perpetuation of their own organization. Hegel upheld Kant’s opposition between Newtonian mechanism and organism, as well as the problem of natural ends. However, when we speak of purpose, we need to distinguish between external and internal purposiveness:

“[t]he notion of end, however, is not merely external to Nature, as it is, for example, when I say that the wool of the sheep is there only to provide me with clothes; for this often results in trivial reflections, […], where God’s wisdom is admired in that He provided cork-trees for bottle-stoppers, or herbs for curing disordered stomachs, and cinnabar for cosmetics” (Hegel, 1830/2004; p. 5, §245).

When Hegel speaks of internal purposiveness and the notion of end, he refers to a logic that pertains to the organization of natural objects:

“[t]he notion of end as immanent to natural objects is their simple determinateness, e.g., the seed of a plant, which contains the real possibility of all that is to exist in the tree, and thus, as a purposive activity is directed solely at self-preservation. […]; the true teleological method—and this is the highest—consists, therefore, in the method of regarding Nature as free in her own peculiar vital activity” (Hegel, 1830/2004; p. 6, §245).

Purposive activity stems not from the projection of intentions, goals, or plans onto the organism. Instead, the organism’s internal purposiveness is grounded in the immanent necessity of self-maintenance: internal purposiveness is self-preservation. The specifically Hegelian twist to the Kantian problem of “natural ends” resides in the assertion of purposiveness immanent to nature. For Kant, purpose remains ultimately at the level of knowledge and explanation; it is “not an objective principle but a merely regulative one, a subjective maxim of the reflecting power of judgment. Therefore it has a value that is not constitutive but simply heuristic” (Michelini, 2012; p. 135). For this reason, from the perspective of Kantian philosophy, we can only conceive of purposiveness “as if” it pertains to nature.

Incidentally, the opposition between external and internal purposiveness is central to Friston’s free energy minimization as well. His framework provides an alternative to normative explanations “in which I, as external observer, write down some condition for optimal behavior, rather than grounding that explanation in the necessary preconditions for the existence for that organism” (Allen and Friston, 2018; p. 2473). By taking the preconditions of the organism as a starting point, “the FEP (free energy principle) provides a normative, teleological essence to the synthesis of biology and information” (Allen and Friston, 2018; p. 2476). In other words, in the case of Friston as well, the organism’s internal purposiveness (“teleological essence”) follows from the necessary condition that has to be met for there to be an organism.

Both in the case of Friston and Hegel, such a necessary precondition is the existence of a boundary between the organism and its surroundings: “the events that ‘take place within the spatial boundary of a living organism’ [Schrödinger] may arise from the very existence of a boundary or blanket, which itself is inevitable in a physically lawful world” (Friston, 2013b; p. 1). In Hegel’s words: “Nature’s formations are determinate [*bestimmt*], bounded [*beschränkt*], and as such enter into existence” (Hegel, 1830/2004; p. 284, §339). In other words, natural objects are to be bounded if they are to exist. As we will see in the next section, the gist of Friston’s framework is that if an organism maintains a boundary, it minimizes free energy.

In the case of Hegel and his discussion of internal purposiveness, it is not enough to assert the importance of the existence of a boundary. If we were to leave it at that we would remain at a determination of the organism in terms of a bounded organization plus the magical power to exert purpose. There is no such “plus” in Hegel; he does not “add something” to mechanical nature; he does not succumb to vitalism. Instead of an addition, Hegel’s solution to the problem of purposiveness consists in subtraction. As we will see below, it is what the organism lacks which gives it purpose or drive. By addressing what it lacks, the organism exerts purpose in the preservation of its organization. There is nothing mysterious about the notion of lack. If we regard an organism as an organized product of nature, then lack is simply a state of disorganization that needs to be addressed (e.g., hunger). Here too, there are striking parallels to Friston’s framework in which the organism continually engages in minimizing the “error” inherent to its internal states, in an attempt to maintain an internal organization.

What makes the organism contradictory is the coincidence of organization and disorganization. “For Hegel, life itself is imbued with contradiction because it is inextricably bound up with what it lacks: its identity is at one with its negation” (Michelini, 2012; p. 133). Hegel’s version of self-organization and self-production is thus a continuous process of self-preservation sustained by contradiction, through which a bounded organization is maintained. In the remainder of this section, we will retrace Hegel’s exposition, which results in the contradictory organism^1^.

### Animal Organism

The entirety of Hegel’s *Philosophy of Nature* forms a succession of stages that progresses from mechanics to organics, culminating in the animal organism. For our purposes, his discussion of the animal organism is particularly relevant, due to its striking similarity with Friston’s free energy minimization. As such, we will focus on Hegel’s discussion of the animal organism:

“The animal organism is the microcosm, the center of Nature which has achieved an existence for itself in which the whole of inorganic Nature is recapitulated [*zusammengefaßt*] and idealized; this will be worked out in the detailed exposition to follow. Since the animal organism is the process of subjectivity, of self-relation in an outer world [*der Äußerlichkeit*], the rest of Nature is therefore here present for the first time as outward, since the animal preserves itself in this relation with the outer world [*zum Äußeren*]” (Hegel, 1830/2004; p. 356, §352).

Almost everything we want to discuss in this section is contained in this passage. Let us unpack it. The advent of living beings for Hegel designates the moment where a natural process has extracted itself from the rest of nature and starts to function on its own terms. What gives animal life a privileged status is that the animal organism maintains a more determinate boundary with its surroundings compared to plant life: “[n]ow the plant is drawn towards the outer world but without truly preserving itself in connection with what is other, and consequently the rest of Nature is still not present for it as outer” (Hegel, 1830/2004; p. 356, §352). While a strong division between plant and animal life is problematic from the standpoint of modern biology (let alone the omission of other forms of life such as single-celled organisms), we are concerned with a simple point that Hegel places great emphasis on: plant life is not separated from its surroundings to the degree that animals are. “The plant, as the first self-subsistent subject […] still has its origin in immediacy” (Hegel, 1830/2004; p. 304, §343). Put simply, this means that the plant cannot “freely determine its place, i.e., *move from the spot*,” and that “its nutrition is not an interrupted process but a continuous flow” (Hegel, 1830/2004; p. 305, §344). This changes with animal life, because “the animal is a true, self-subsistent self which has attained to individuality, it excludes and separates itself from the universal substance of the earth which is for it an outer existence” (Hegel, 1830/2004; p. 355, §351). The animal maintains delineated break with its surroundings. While plant life “still has its origin in immediacy,” the animal organism has suspended the immediacy of its surroundings.

In the maintenance of itself as a distinct entity (“for itself”), the animal forms a recapitulation and “idealization” of inorganic nature. “Idealization” is what Malabou translates as “contraction”; around which Hegel’s entire *Philosophy of Nature* is organized: “Hegel intends to show how the living organism summarizes everything that precedes it: inert matter, the elements, chemical processes, all the constitutive moments which are dialectically conjoined” (Malabou, 1996/2005; p. 59). But the process of contraction goes further. As we will see further on, contraction designates the process through which the organism reproduces its constitution, in the broadest possible sense. The name for this constitution is habit.

In the suspension of immediacy; in the maintenance of a boundary, the animal organism has replaced natural immediacy with a second immediacy that is posited by the organism itself. This substitution is the “characteristic of habit,” through which nature is redoubled and as such starts to appear as “second nature” (Malabou, 1996/2005; p. 37–38). The living organism, and the animal in particular, maintains a minimal difference between itself and its immediate surroundings. In the maintenance of this difference, the animal perpetuates itself as a habitual structure. The structural elements that constitute the animal organism—through which it contracts habit—are what Hegel calls “Shape [*die Gestalt*]” and “assimilation [*die Assimilation*]” (Hegel, 1830/2004; p. 356, §352).

The animal organism’s shape [*Gestalt*] is in turn comprised of three constitutive moments: (1) “its simple, *universal being-within-self* in its externality [*allgemeines* *Insichsein in seiner Äußerlichkeit*]”; “it is an undivided identity of the subject with itself—sensibility”; (2) “a capacity for being stimulated from outside and the subject’s own reaction outwards to the stimulation—irritability”; and (3) “the unity of these moments, the *negative* return to itself from its relation with the outside world, and, through this, the production [*Erzeugung*] and positing [*Setzen*] of itself as a singular—reproduction, which is the reality and the basis of the first two moments” (Hegel, 1830/2004; p. 357, §353; see Michelini et al., 2018, Figure 1, for a full schematic overview of the animal organism in Hegel’s *Philosophy of Nature*).

As is characteristic of Hegel’s philosophical exposition, these three moments can be regarded separately, in which case they remain abstract, but only when considered together as a single movement do they do justice to the totality of the process constituting the animal organism:

“Reproduction passes through sensibility and irritability and absorbs them; it is thus derived, posited universality which, however, as self-producing [*das Sichproduzieren*], is at the same time concrete singularity. It is reproduction which is first the whole—the immediate unity-with-self in which the whole has at the same time entered into relationship with itself” (Hegel, 1830/2004; p. 358, §353).

In other words, reproduction here does not refer to replication, but to “self-production, or the active conservation of a self-produced identity” (Marques and Brito, 2014; p. 92). The totality of the movement of reproduction includes sensibility and irritability. Let us start with sensibility, which designates the identity of the animal organism with itself: the “the sentient creature [*das Empfindende*]” (Hegel, 1830/2004; p. 358, §354). When considered in isolation, the “system of sensibility” is constituted by “the extreme of *abstract* self-reference” (Hegel, 1830/2004; p. 357, §354), which simply designates the rudimentary differentiation of the animal with regards to its immediate surroundings, whereby it maintains a minimum of autonomy.

However, the animal not only exists in isolation: the system of sensibility is differentiated outwards through the nervous system, on account of which the animal has “an inward and outward reference—the sensory and motor nerves, respectively” (Hegel, 1830/2004; p. 359, §354). Indeed, it is through the nervous system that the animal is sensitive to (outside) influences:

“The moment of difference in sensibility is the nervous system which is directed outwards and is involved in external relationships: sensation [*Empfindung*] as determinate—either as immediately posited from outside or as self-determination [*Fühlen oder Selbstbestimmung*]. The motor nerves mostly start from the spinal cord, and the sensory nerves from the brain: the former are the nervous system in its practical function, the latter are that system as receptive of determinations, and to this the sensory organs belong” (Hegel, 1830/2004; p. 363, §354).

The first thing to note in this passage is that Hegel introduces a rudimentary circular process (“self-determination”) through which the organism can determine its own sensations on the basis of its motions. In the next section, we will see that such circularity is crucial within Friston’s free energy minimization. The second thing to note is that, as the animal is “receptive to determinations,” it not only maintains a break with its surroundings, but it also maintains a break with itself. As the animal “enters into relationship with itself,” the animal “has itself for its object” (Hegel, 1830/2004; p. 353, §351). This self-relation, self-differentiation, or self-feeling is sensation, which is the “absolutely characteristic feature [*Bestimmung*] of the animal” (Ibid.). As we will see later on, free energy minimization also functions on the basis of the incorporation of sensation or differentiation, in the form of anticipatory “error.”

The movement of self-determination not only pertains to differentiation inwards; the animal also differentiates itself outwards: “the subjectivity of the animal is not simply distinguished from external Nature, but the animal distinguishes itself from it; and this is an extremely important distinction, this positing of itself [*Sichsetzen*] as the pure negativity of *this* place, and *this* place, and so on” (Hegel, 1830/2004; p. 354, §351). Which brings us to the second moment: “irritability is just as much a capacity for being stimulated by an other and the reaction of self-maintenance against it, as it is also, conversely, an active maintenance of self” (Hegel, 1830/2004; p. 359, §354). In other words, building on top of the capacity for self-differentiation or sensation, irritability designates the organism’s capacity to react to, or act on, its sensations. In the combination of the self-determining loop of sensibility, and the capacity to react and maintain itself in irritability; we have the necessary ingredients to complete the overarching loop that constitutes the third moment: reproduction. In passing through both sensibility and irritability, the animal continually (re-)produces and maintains itself as a distinct entity. Let us continue to show the totality of this movement operates in the next stage Hegel distinguishes.

The organism’s reproductive movement actualizes in relation to its surroundings, through which the organism appropriates these surroundings:

“Now since the organism is directed towards the outer world as well as being inwardly in a state of tension towards it [*innerlich dagegen spannt*], we have the contradiction of a relationship in which the outer must be sublated [*aufgehoben*]. The organism must therefore posit what is external as subjective [*das Äußerliche als subjektiv setzen*], appropriate it, and identify it with itself; and this is assimilation” (Hegel, 1830/2004; p. 381, §357).

In other words, as the animal necessarily stands in relation to its surroundings; its reproduction takes the form of perpetual activity, through which the animal assimilates nature external to itself. The animal’s relation to food makes up the most elementary example of this process. As the organism strives to overcome the deficiency of hunger, it passes through the three stages of sensibility, irritability, and reproduction in the corresponding sequence of hunger, ingestion, and digestion (Michelini et al., 2018; p. 8). As such, the organism reproduces itself through the ingestion and subsequent digestion of food.

The animal’s capacity for sensation permits the experience of tension: the “self-feeling of the individuality is also directly exclusive and in a state of tension with a non-organic nature which stands over against it as its external condition and material” (Hegel, 1830/2004; p. 380, §357). And while this material may be employed to alleviate states of lack or deficiency such as hunger, the overcoming of deficiency offers only temporary respite: “the animal perpetually returns from its satisfaction to a state of need” (Hegel, 1830/2004; p. 391, §362). In other words, the animal only temporarily overcomes its deficiency and necessarily revisits and maintains a state of tension with its surroundings.

While hunger is the most readily available example of this process, the simplicity of the example should not deceive us: its basic logic is “particularized in an infinite variety of ways” (Hegel, 1830/2004; p. 388, §360). While the example of hunger is intuitive, it does not go far enough, because in order to speak of plasticity in terms of simultaneous reception and production of form, we need a process that not only alleviates states of need, but we need a process able to reconfigure the organism’s habitual structure itself. Indeed, the generalized logic of “digestion” is what we are dealing with in the process of assimilation, where the animal contracts and posits its surroundings as part of its own habitual structure. The result of this continuous abstraction-contraction-recapitulation is “literally, habitus, at once the internal disposition and the general constitution of the organism” (Malabou, 1996/2005; p. 59). The “general constitution” of the organism refers to the recapitulation of inorganic nature that came before it, while the organism’s “internal disposition” signifies the organism’s contracted habitual structure. As we will see later on, it is the generalized logic of perpetual contraction that we find in Friston’s free energy minimization, in the sense that the configuration of an anticipatory model “feeds off” its surroundings.

### Contradiction and Anticipation

We began with the organism understood as a self-enclosed entity; an abstract “system of sensibility” which is subsequently differentiated outwards on account of the nervous system. In the second moment of irritability, the organism is not only receptive to stimulation but reacts to such stimulation by engaging in active self-maintenance. Through these moments of sensibility and irritability, the organism embodies a self-productive loop in which its surroundings are assimilated. With these two stages of shape [*Gestalt*] and assimilation, we passed from an abstract to a relational understanding of the organism. We now have the necessary ingredients to take the third and final step in order to show what makes the organism contradictory.

If we emphasize the organism’s autonomy, the problem is that the relation of the organism to what lies outside it remains an external relation. Its conception remains abstract; as if the organism has a choice to engage or not with its surroundings. If we take a step further, we are forced to admit that the organism “always already” stands in relation to its surroundings, as it is internally strung in opposition to it. However, if we emphasize the organism’s perpetual relationship to its surroundings, we risk losing the very condition for its existence as a distinct entity: the boundary between organism and surroundings. The difficulty resides in conceiving of the organism as both abstract and relational. More precisely, the point is not to choose either an abstract or a relational conception of the organism. Rather, the point is to see how the organism’s relation to and dependence on its surroundings is simultaneously constitutive for the organism understood as a distinct or an “abstract” entity. Therein resides the organism’s contradiction:

“Although common thought has it that need indicates dependence on something else, in reality, in a paradoxical way, it is a manifestation of independence: in fact water and food would be totally indifferent to the living being and they would not be able to have a ‘positive’ relation with it if the living being was not, for Hegel, ‘the possibility of this relation’.” (Michelini, 2012; p. 137).

Said differently, the paradox is that the organism’s relation to something outside itself is simultaneously the guarantor for the minimum of its autonomy. There is no choice to be made here between abstract and relational: the organism itself is subject to the tension between independence and dependence in the perpetuation of its life. For there to be a relationship of dependence between organism and surroundings, there needs to be an independent organism in the first place, in the sense of a distinct entity that is able to engage in a relationship of dependence. We can also turn this around: the only way for the organism to conserve and perpetuate itself as an independent entity is to engage in a continuous relationship of dependence with its surroundings, through which the organism assimilates external nature. This relationship of dependence, in turn, is continually reinvigorated by the organism’s recurring state of need. In this sense, the tension between independence and dependence is operative at the level of the organism itself. Insofar as the organism maintains a boundary between itself and its surroundings, it perpetually revisits a state of tension in need of alleviation. As such, sustained tension is concomitant with the existence of a boundary. As we will see below, this tension internal to the organism’s organization is the sustained contradiction which serves as a precondition for the organism’s existence concurrent with the boundary that constitutes its life.

With the organism’s constitutive contradiction, we return to the problem of internal purposiveness from the start of this section. For Hegel, the organism’s loop of self-determination is sustained by the contradiction it sustains, which gives the organism its internal purposiveness: “[n]eed and drive are the readiest examples of [internal] purpose. They are the felt contradiction, as it occurs within the living subject itself; and they lead into the activity of negating this negation […]” (Hegel, 1830/1991; p. 281, §204). The living organism (subject) feels contradiction, and its activity is directed towards overcoming this contradiction by getting rid of (negating) the feeling of need: the process begins with “the feeling of *lack* [*Mangel*], and the urge [*Trieb*] to get rid of it [*ihn aufzuheben*]” (Hegel, 1830/2004; p. 385, §359). But because the organism’s contradiction is constitutive for its existence, there is no definite escape from it as long as the organism is alive: “[t]his contradiction, that they are and are not, […], manifests itself as a perpetual process” (Hegel, 1830/2004; p. 377, §356). In its perpetual attempts at overcoming its needs, the animal engages in “activity of deficiency” (Michelini, 2012; p. 137).

Therein resides the paradoxical status of living beings: the organism harbors at once the maintenance of its own identity, as well as the negation of this identity. “The defect [*Mangel*] in a chair which has only three legs is in us” (Hegel, 1830/2004; p. 387, §359). The organism functions like a three-legged chair that stays upright but continually slants into the direction of its missing fourth leg. As Francisco Varela put it: “If we invert our perspective, this constant bringing forth of signification is what we may describe as a permanent lack in the living: it is constantly bringing forth a signification that is missing, not pregiven or pre-existent” (Varela, 1997; p. 80). In the continuous attempts at suspending its needs, the organism reproduces itself by contracting its surroundings. It is in this sense that the organism organizes itself around the contradiction that constitutes its existence. Insofar as this organization endures, the name for this organization is habit. As we will see later on (“Contradiction Again” section), in mobilizing this contradiction immanent to the organism, Hegel anticipated contemporary discussions surrounding the appropriation of Friston’s framework in terms of cognitivism and enactivism.

What makes Hegel’s philosophy difficult to follow is that he forces us to retrace numerous processes separately that he subsequently ties in together. What makes it outright frustrating is that the tension within the exposition itself is transposed into the object under study. The result is that by the end of the exposition, our initial starting point has been problematized. This is what Helmholtz missed when he dismissed Hegel for starting with the “Hypothesis of Identity” (von Helmholtz, 1995; p. 79). In our case, the abstract conception of the animal organism as a self-enclosed entity is transformed through a relational designation, into an entity that appears as inherently contradictory. In this sense, the same circular structure of the organism’s self-production also pertains to Hegel’s philosophy itself. His philosophical exposition takes the form of a circular process, which, at the end of the circle, has retroactively undermined its own starting point. Again, in the case of the organism, the point is not to exchange an abstract conception for a relational one, from where the organism is engaged in continuous exchange with its surroundings. The organism is both abstracted from its surroundings as it maintains its boundary, and it stands in constant relation to these surroundings. The point is to recognize that the tension between abstract and relational is actualized and operative at the level of the animal organism itself in the form of recurring lack. It is this contradiction between habitual structure and lack, or organization and disorganization, which gives rise to the organism’s internal purpose or drive.

Hegel’s animal organism thereby goes beyond (Newtonian) mechanism under the immediate influence of external nature: the “organism is no longer ‘determined by external causes’ but irritated by external forces” (Marques, 2016; p. 128). The suspension of the immediacy of external nature functions on the basis of the boundary that separates the organism’s “second nature” or habit. It is on account of the animal’s contracted habitual structure and the deficiencies that mark this structure, that the animal operates on the basis of its own purposiveness: it “is only as this self-reproductive being, […], that the living creature *is* and *preserves itself*; it only is, in making itself what it is, and is the antecedent End which is itself only result” (Hegel, 1830/2004; p. 356, §352). How does the contraction of habit relate to the notions of plasticity and dialectics?

Both notions designate not a property or attribute of the living organism, but they are two ways of approaching the living organism *as a process*. This is crucial, because for Hegel, “[s]tructure, as alive, is essentially process” (Hegel, 1830/2004; p. 377, §356). As we briefly stated in the introduction, plasticity in Hegel designates “a capacity to receive form and a capacity to produce form” (Malabou, 1996/2005; p. 9). The process we have been describing so far is plastic: in the contraction of its surroundings, the organism is not only formed by its surroundings, but it produces its own form in the process. The moment forces external to the organism cross over onto the terrain of the organism, the organism posits and molds external nature as part of itself.

The process of plasticity is simultaneously dialectical, because the operations which constitute it, “the seizure of form and the annihilation of all form, emergence and explosion, are contradictory” (Malabou, 1996/2005; p. 12)^2^. The formation of habit does not pertain to a stable entity to which habits are added and subtracted like attributes. The formation of habit *is the formation of the organism as such*: “[h]abit is there not only as a particular momentary satisfaction; rather I am this habit. It is my universal mode of being—what I am is the totality of my habits. I can do nothing else, I am this” (Hegel, 1994/2007; p. 153). It is because habit constitutes the organism that every change to its formation implies a simultaneous deformation. This coincidence of contradictory moments is what makes the process of plasticity, the contraction of habit and the perpetual reproduction of the organism, dialectical.

It is also the process of plasticity which makes the animal anticipatory: “[n]eed, appetite, desire, the accumulation of such retentions and expectations, are themselves proof of the fact that the animal is concerned to ensure the perpetuation of its own life” (Malabou, 1996/2005; p. 64). In the reproduction of itself, the animal not only maintains a relation to itself and its surroundings, but it also stands in relation to its future: “it is the structure of anticipation through which subjectivity projects itself in advance of itself, and thereby participates in the process of its own determination” (Malabou, 1996/2005; p. 18). The perpetual reappearance of need and the drive to overcome need, indicate the animal’s anticipatory disposition. More precisely, they indicate that the animal itself is simultaneously a rudimentary *structure and process* of anticipation. In the perpetual process of its own restructuring, the animal posits the presuppositions of its own anticipations. Hegel and Friston share the same starting point: a boundary between organism and surroundings, but can we conceive of Friston’s anticipatory brain as a process that is at once plastic and dialectical?

## States of Anticipation

### Anticipatory Brain

The breakthrough of Friston’s free energy minimization stems from the unconventional answer it provides to a simple question: what does the brain do? From the perspective of Friston’s framework, neural functioning is subordinated to the overall imperative of an organism to maintain a boundary (Allen and Friston, 2018; Ramstead et al., 2018). In the process of boundary-maintenance, the brain is caught in a continuous process of anticipation in terms of both perception and action. It is this approach to the brain that has been well received in the philosophy of mind (Clark, 2013, 2015; Hohwy, 2013, 2016; Wiese and Metzinger, 2017; Bruineberg et al., 2018). The conception of the brain as an anticipatory or predictive structure forms the first substantial challenge within neuroscience to the conception of the brain in terms of the “computer metaphor.” In the traditional view, we approach the brain as a computer that primarily processes information from outside; similar to the way data is fed into a computer, which is subsequently processed and potentially retained for later use. While such bottom-up, stimulus-driven processing undoubtedly makes up an important part of what the brain does, anticipatory accounts suggest that the importance of such bottom-up processing has been overstated. From the perspective of these accounts, a (matured) brain is engaged to a much greater degree in the prediction or anticipation of its input. Instead of processing influences from outside, the brain first and foremost constitutes its reality on the basis of what it learned to expect, onto which influences from outside intrude.

It is not the case that the anticipatory perspective introduces top-down influences into the way we view the brain. The traditional view also assigned importance to top-down influences in the form of memory, cognitive control, attention etc. In the traditional view, however, such top-down processes remained secondary in dealing with influences from outside. From the anticipatory perspective, external influences become secondary to anticipatory states. What is so radical about this conception is that it turns the traditional view of neural functioning on its head: external influences get caught up in the primary process of the brain’s anticipatory activity. As we will see below, the brain’s main locus of activity becomes the minimization of the errors stemming from its own anticipations, through which it iteratively settles on anticipations of the future. The brain’s imperative becomes to explain away the mismatch (prediction error) between its anticipations (top-down) and its actual input (bottom-up), across the cortical hierarchy. This mismatch, in turn, serves as a driver for change in the structure that generates future anticipations. It is important to emphasize that such anticipations do not pertain to perception alone. The perceptual aspect of anticipation stands in the service of action. In addition, the input that potentially perturbs the brain’s anticipations is not only passively received from outside, but can equally be elicited by the structure’s own actions (Adams et al., 2013). That is, through action, the brain can actively change its outer world into a state the brain itself anticipated; minimizing prediction error in the process.

An important advantage of Friston’s framework is that it stays close to the anatomical and physiological organization of the brain. For example, the primacy of top-down predictions over bottom-up input fits well with findings showing that top-down connections are both more divergent and abundant than bottom-up connections (Friston, 2005). In addition, the framework is able to adhere to the local computational constraints that individual neurons are subject to Bogacz (2017). The criticism that free energy minimization is unfalsifiable misses the point: Friston’s framework does not stand in contrast to traditional neuroscience at the level of testable theories; it is opposed to traditional neuroscience precisely at the level where *traditional neuroscience itself is unfalsifiable*. As in the case of the computer metaphor.

The opposition between anticipatory accounts and traditional neuroscience touches on two problems discussed earlier: how to do justice to the active organizational capacity that pertains to the brain’s plasticity? And how to conceive of the “self-propagating formative power” that pertains to living organisms? In answering these questions, a simple action-reaction (or stimulus-response) account seems inadequate, but we simultaneously need to avoid adding external purpose to mechanical nature. The often-employed way out of this deadlock in biology is to approach living organisms as dynamical systems (Von Bertalanffy, 1950; Ashby, 1960; Maturana and Varela, 1972/1980; Levins and Lewontin, 2006). From the perspective of these approaches, living organisms are conceived as systems that maintain themselves through a process of self-organization and self-production (*autopoiesis*). It is such a process that Friston formalizes with free energy minimization, which attempts to capture brain function in the broadest possible sense. In the remainder of this section, we will attempt to elucidate Friston’s framework (“Tension Redoubled” section), in order to bring out its convergence with Malabou’s reading of Hegel (“Contradiction Again” section).

### Free Energy Minimization

Free energy minimization is so broad in fact, that it does not pertain solely to brain function; it is a framework pertaining to living systems as such. For this reason, Friston not only reopens the question “what does the brain do?,” but the same goes for the even broader question: what is life? The specific organization of the brain is not primary, but rather “the consequence of, or requirement for, this fundamental imperative [of free energy minimization]” (Friston, 2013a; p. 212–213). As such, we will speak primarily of the organism from here on out, which subsumes the anticipatory brain. Friston suggests:

“that biological self-organization is not as remarkable as one might think—and is (almost) inevitable, given local interactions between the states of coupled dynamical systems. In brief, the events that ‘take place within the spatial boundary of a living organism’ may arise from the very existence of a boundary or blanket, which itself is inevitable in a physically lawful world” (Friston, 2013b; p. 1).

In order to make sense of Friston’s framework, we have to adopt the language of statistical physics. This means that we approach the organism as a system that stands in relation to its local surroundings or environment. Because the system is always already embedded in this relation, it necessarily lacks a complete overview of both its own possible states, as well as the possible states of its local surroundings. Free energy minimization comes down to a computational problem in which the organism attempts to minimize the mismatch between its internal states on the one hand and the (inferred) external states of its surroundings on the other. What is a state? “Formally speaking, the state of a system corresponds to its coordinates in the space of possible states, with different axes for different variables […]” (Friston, 2018a; p. 2). Put simply, a multitude of states forms the structural configuration of the organism: “the repertoire of physiological and sensory states in which an organism can be is limited, and these states define the organism’s phenotype” (Friston, 2010; p. 127).

With the notion of “state” we touch on a common thread with regards to Hegel. Hegel’s notion of “habit” stems from his reading of Aristotle’s *De Anima*. The etymology of the word “habit” leads back to the Latin *habere*: “a way of having,” and the Greek verb. This verb means “to have,” but as soon as it is followed by an adverb, it changes its meaning to include “the *state* of being in one way or another” (Malabou, 1996/2005; p. 37; italics ours). We thus find here an additional indication that both lines of thinking are closer than they may appear: both “state” and “habit” refer to the configuration of the anticipatory organism.

The internal states of the system and the external states of its surroundings are separated by the organism’s intermediary layer comprised of sensory and action states. For the organism, the problem of maintaining itself consists in coordinating the (dis)concordance between different kinds of states. From a perspective outside the system, the difficulty in understanding how the coordination of these states unfolds, resides in that we are dealing with a circular structure, where each kind of state presupposes another. A sensory state presupposes the evocation of said state, either from outside or by the organism itself. If the organism acted on its own sensory states, such action presupposes an anticipation on the basis of which to act. Such an anticipation, in turn, requires internal states on the basis of which to anticipate. And finally, the existence of an internal organization necessitates the existence of a delineated organism in the first place. We are thus dealing with a loop in which internal states, action states, external states, and sensory states mutually implicate each other. In other words, the existence of a boundary between organism and surroundings is a necessary precondition for the process to take place; the boundary itself “induces a circular causality” (Allen and Friston, 2018; p. 2474).

The proximity to Hegel is clear. In his work as well, the discussion of the animal organism presupposed differentiation: a bounded organization that sustains a break with external nature. Insofar as the animal organism is alive it upholds a minimum of autonomy by maintaining a boundary between itself and its surroundings. In addition, animal life for Hegel is also caught in a loop of sensation and motion, which gives the organism the capacity for self-determination. In the case of Friston, the idea is that if a separation between internal and external states exists, then the system must minimize free energy. Why?

The logic is as follows: out of all possible states the system can be in, there is a relatively small number of states that sustain its life. This implies that, if an organism endures, it will necessarily frequent a limited number of states that lie within its physiological bounds. The maintenance of a limited number of states implies that the system will need to try to minimize surprises with regards to its own states. For example, “a fish out of water” would be in a surprising state (both emotionally and mathematically)’ (Friston, 2010; p. 127). Under the assumption that the system limits its own states, “the long-term average of surprise is entropy. This means that minimizing free energy—through selectively sampling sensory input—places an upper bound on the entropy or dispersion of sensory states” (Friston, 2012; p. 2). The maintenance of such a limited number of states designates the way biological systems resist “the dispersive effects of fluctuations in their environment” (Friston, 2012; p. 1). In other words, the minimization of free energy designates how an organism resists its surroundings and thereby maintains an internal organization.

The placement of an upper bound on surprise simultaneously implies its obverse; namely, “systems that minimize free energy also maximize a lower bound on the evidence for an implicit model of how their sensory samples were generated” (Friston, 2012; p. 2). This “implicit model” is the anticipatory structure generating the organism’s anticipations. Such a generative model “aims to capture the statistical structure of some set of observed inputs by tracking (one might say, by schematically recapitulating) the causal matrix responsible for that very structure” (Clark, 2013; p. 2). Taken together, free energy minimization implies that the system limits the states it frequents (by placing an upper bound on the surprise of these states), and thereby maximizes the evidence for the “implicit model” that it is. As in the case of Hegel, this model is constituted in a continuous process of contracting (schematically recapitulating) the organism’s surroundings.

But what is free energy? What does the organism minimize exactly? Friston says:

“Free-energy is an information theory quantity that bounds the evidence for a model of data. Here, the data are sensory inputs and the model is encoded by the brain. More precisely, free-energy is greater than the negative log-evidence or ‘surprise’ in sensory data, given a model of how they were generated. Crucially, unlike surprise itself, free-energy can be evaluated because it is a function of sensory data and brain states. In fact, under simplifying assumptions […], it is just the amount of prediction error” (Friston, 2009; p. 293).

What sets Friston’s framework apart from just another formal description of the organism’s behavior is that it formalizes how the organism (including the brain) is able to maintain itself *from the perspective of the system itself*. This starting point is crucial because it takes seriously how the system lacks access to the distribution of all possible states external to it. It cannot know all possible configurations of its surroundings. As a matter of fact, the system does not have access to external states at all, apart from the sensory states these external states elicit. In this sense, external states are “hidden.” It is because external states and their distribution are only indirectly accessible that the organism is unable to minimize surprise directly. Instead, the organism engages in the minimization of an upper bound on surprise, which is a problem the organism can solve, because it relies not on having access to the distribution of possible external states, but only on internal states (the model in the citation above) and elicited sensory states (the data). The system minimizes this upper bound on surprise either by changing its sensory input through action; a process Friston calls active inference; or by altering its internal anticipatory model: perceptual inference. On the one hand, “action is the only way to underwrite an upper bound on the entropy of sensations. On the other hand, perceptual inference is the only way to inform action” (Allen and Friston, 2018; p. 2477). For example, if the system cannot make out what caused its sensations, in addition to inferring a probable percept given the context, another option is to move closer in order to get a better look. In other words, the system has two ways of minimizing free energy; it minimizes the error of its own anticipations through perceptual and active inference.

In the process of inference, anticipatory or prediction “errors” refer to the deviation of sensory data from the predictions or anticipations formulated by the system. For this reason, it is important to emphasize that these “errors” are “wrong” only in relation to the anticipations that arise from the system itself. The standard by which an “error” is determined is strictly immanent to the system. How the system deals with prediction error is important, because these errors determine both the system’s configuration, as well as the actions the system engages in. On the one hand, if these errors are not taken serious enough, the system is at risk of detaching itself from the demands of its surroundings. On the other hand, if these errors are taken too seriously, the system may find itself engrossed by every insignificant change in scenery. In order to cope with anticipatory error, the system’s challenge is not only to infer its external states, but the system also needs to determine the degree of certainty or precision of its inferences. The perspective free energy minimization subscribes to “suggests that there are only two sorts of things that need to be inferred about the world; namely, the state of the world and uncertainty about that state” (Friston et al., 2012a; p. 2). To connect with what we said above: uncertainty is another way to conceive of entropy; to minimize free energy means to resist being overwhelmed by uncertainty: “[o]ur objective, given a model (brain), *m*, is to minimize the average uncertainty (entropy) about some generalized sensory states” (Feldman and Friston, 2010; p. 5). In order to constrain the uncertainty of its states, the system needs to estimate the precision of the disruption (prediction error) of its anticipatory states. If such disruption is estimated to be precise, it will exert more influence on future anticipations compared to imprecise disruption. In the interplay between anticipation and disruption, the dominance of either is dependent on the precision afforded to both. This enduring conflict amounts to how something is perceived and what is to be done.

An important implication of Friston’s framework is that it gives a purely formal description of what the system does in terms of boundary preservation. In doing so, the framework does not provide an externally imposed normative account of “optimal” behavior in terms of, for example, reward maximization. While Friston’s framework prescribes that the system minimizes free energy; *what* this minimization entails fully depends on the configuration of the anticipatory system. In other words, the specifics of free energy minimization are fully dependent on the contracted internal constitution of the anticipatory organism, and the surroundings in which this habitual structure dwells. For that reason, the only normativity involved in free energy minimization is that it emphasizes “the necessary tendency of living organisms to resist the second law of thermodynamics; i.e., to maintain an internal structure or dynamics in the face of constant change” (Allen and Friston, 2018; p. 2473). Friston’s approach to neural functioning thus describes and formalizes how, once a state of relative equilibrium is acquired—to the point where a maintainable boundary is established—the system organizes and produces itself through a perpetual loop of sensation and action.

### Plasticity? Dialectics? Contradiction?

The etymological proximity of “habit” and “state” indicates that both in the case of Hegel and Friston, what is at stake is the organization of an anticipatory structure. In both cases, the organization of this structure takes the form of a continuous process. Given a boundary, there is a process of boundary-maintenance. And given the process of boundary-maintenance, the structure constituted by the boundary is maintained. As such, the only stability that pertains to this structure consists in the perpetual maintenance of a separation between inside and outside. In the active process of boundary-maintenance, an organism under free energy minimization is no longer a passive processor of external influences, but does this mean that free energy minimization adheres to the notion of plasticity, as understood by Malabou?

Plasticity for Malabou designates the capacity to receive as well as the capacity to produce form. In the case of Hegel, we saw that the organism receives form from external nature, but it also produces form by positing external nature as part of its own organization. This process of self-production takes the form of a self-referential process through which the organism participates in its own determination. In the case of Friston’s framework, we are dealing with exactly the same process. As we have seen, the organism minimizes free energy by actively limiting the states it occupies through a circular process implicating both sensation and action. As such, free energy minimization constitutes a loop in which the reception of form (sensation) and the production of form (action) are implicated in the same circular process. However, while the self-determination of sensation is a necessary requirement for the self-production of form, in order to speak of plasticity in a Hegelian sense we need a process which captures the reverberation of self-differentiation able to alter the contracted constitution of the organism’s anticipatory structure. Does free energy minimization capture this process? Clearly it does; as the organism engages in perceptual and active inference, the anticipatory model itself is continually altered in confrontation with the disruption of the anticipations generated by this model. Under free energy minimization, the reception and production of form are implicated in the same process: “learning and perception are two sides of the same coin”: “perceptual inference (i.e., neurodynamics) and learning (i.e., neuroplasticity) are in the game of optimizing the same thing; namely, model evidence or its variational equivalent (i.e., free energy)” (Friston, 2018b; p. 1020–1021). The only difference is that Friston’s distinction between neurodynamics and neuroplasticity, fall under the Hegelian notion of plasticity. As such, free energy minimization is a plastic process. In this sense, Friston’s framework moves beyond the one-sided conception of plasticity criticized by Malabou, but is this process dialectical?

What makes the process of plasticity dialectical for Malabou is that in it, contradictory operations coincide, in the sense that “the seizure of form and the annihilation of all form, emergence and explosion, are contradictory” (Malabou, 1996/2005; p. 12). The reception and production of form are not only subsequent stages in the formation of the organism, but both capacities actualize simultaneously. Every reception of form implies the simultaneous production of form as the organism incorporates the influences it undergoes. Put differently, whenever the organism converges on a state, the organism’s overall state by definition encloses anticipation and the disruption of anticipation in the form of anticipatory error. In this sense, as in the case of Hegel, Friston’s anticipatory organism “has itself for its object” and maintains a break not only with its surroundings, but with itself as well. It is only in the coincidence of the anticipatory organism’s structure (identity) and its disruption (“error”) that this structure is altered in anticipation of what to expect next: “the threat of the explosion of form structurally inhabits every form. All current identity maintains itself only at the cost of a struggle against its autodestruction: it is in this sense that identity is dialectical in nature” (Malabou, 2004/2008; p. 71). In other words, the coincidence of (perceptual) inference and learning stems from the coincidence of anticipation with its negation. As such, the organism is simultaneously and necessarily subject to deformation in the process of its own formation. The convergence of Hegel and Friston shows how Friston’s free energy minimization is a modern-day instantiation of Hegel’s dialectical process of plasticity. Indeed, how “it is Hegel who will have discovered before its discovery the plastic materiality of being: that free energy, whether organic or synthetic, which circulates throughout in each and every life” (Malabou, 1996/2005; p. 193).

And yet, it is not enough to designate the formation-deformation of the organism’s structure-process. Hegel’s *Philosophy of Nature* does not stop there. We need to ground the unrelenting continuation of this process. Said differently, we need to ground the anticipatory organism’s internal purposiveness. How? We saw that Hegel’s designation of the organism ended with the primacy of contradiction constitutive of the organism’s life: in passing through the tension between abstract and relational, we saw that the organism itself sustains contradiction between habitual structure and deficiency. We need to show how the movement of plasticity is sustained by “the contradictory tension between particular determinacy and its dissolvement into the universal” (Malabou, 1996/2005; p. 12). It is here where Friston, and the appropriations of his framework in terms of cognitivism and enactivism, do not go far enough.

## From Tension to Contradiction

The first question we set out to answer was the following: is free energy minimization a formalization of a dialectical process of plasticity, as understood by Malabou as the capacity both to receive and to produce form? Above we saw that this is indeed the case: both Malabou and Friston capture a self-productive process which simultaneously implicates *formation* as well as *deformation*. That brings us to the second question we posed: can we enact the Hegelian shift from tension to contradiction with regards to free energy minimization? What tension are we referring to?

There are two tensions that pertain to free energy minimization. The first is theoretical: the tension between cognitivism and enactivism; seclusion and openness. Cognitivism and enactivism are two theoretical frameworks that fundamentally differ in their approach to the mind and brain. Cognitivism dates back to the cognitive revolution of the 1950’s, in which the emphasis shifted from behaviorism to the way the mind processes information (e.g., see Miller, 2003). Enactivism is a more recent approach which builds on ecological psychology, embodied cognition, and situated cognition. It is often contrasted with cognitivism, because enactivism places great emphasis on the dynamic interaction between brain, body, and environment, beyond mere information-processing (e.g., see Thompson and Varela, 2001). As we will see below, the appearance of Friston’s framework differs substantially, depending on the perspective we adopt.

The second tension pertains to the organism itself: under free energy minimization, the anticipatory organism is subject to the unsolvable imperative to minimize free energy, in that it continually sustains anticipatory error. Our wager is that these tensions overlap: the tension between seclusion and openness *is the unsolvable tension of free energy minimization*. We will begin with the theoretical tension (“Seclusion and Openness” section), after which we will move on to the unsolvable imperative of Friston’s framework (“Tension Redoubled” section). Finally, we will attempt to enact the shift from tension to contradiction with regards to free energy minimization (“Contradiction Again” section).

### Seclusion and Openness

The theoretical struggle surrounding the appropriation of Friston’s framework revolves around “the tension between internalist and externalist approaches” (Allen and Friston, 2018; p. 2478). This tension revolves around the following question: is the organism separated or secluded from the surroundings in which it dwells, or should the emphasis lie with the organism’s embeddedness in and perpetual openness to these surroundings?

The first position is defended most forcefully by Jacob Hohwy:

“All perceptual and active inference happens in an interplay between the evidence to the system, that is, activity at the sensory epithelia, and the predictions generated under the overall model in the brain. This creates a sensory blanket—the evidentiary boundary—that is permeable only in the sense that inferences can be made about the causes of sensory input hidden beyond the boundary” (Hohwy, 2016; p. 265).

From this perspective, the emphasis lies fully with the seclusion of the internal anticipatory model within the confines of the skull. All of the organism’s activity (bodily or otherwise) stands in service of the optimization of this model. As such, free energy minimization becomes “more neurocentrically skull-bound than embodied or extended, and action itself is more an inferential process on sensory input than an enactive coupling with the body and environment” (Hohwy, 2016; p. 259).

The second position—emphasizing the brain and body’s embeddedness/perpetual openness—is formulated succinctly by Jelle Bruineberg and Erik Rietveld:

“The FEP [free energy principle] implies a deep connection between the dynamics of the brain-body-environment system and the neurodynamics. […] The function of the generative model is therefore not to provide the agent with a representation of the dynamical structure of the environment *per se*, but rather to steer its interactions with its environment in such a way that a robust brain-body-environment system is maintained” (Bruineberg and Rietveld, 2014; p. 7).

From the enactivist position, the skull-secluded internal model stands in service of the organism’s selective openness towards the affordances provided by the environment. Due to this enduring relation between the organism and its surroundings, the “organism does not need to have a model of its niche, but rather the claim is that the structure of the niche is reflected in the structure of the skilled embodied organism” (Bruineberg and Rietveld, 2014; p. 8). In other words, the organism “does not have a model of its world—it is a model” (Friston, 2013a). How to resolve the tension between cognitivism and enactivism?

Not only do both positions have merit, but they follow logically from free energy minimization, and neither are naïve. The enactivists recognize the requirement for a distinctive entity in order for there to be a “brain-body-environment system”: “[o]ne might object that there is still a non-trivial boundary separating the system from its environment […]. We agree […]” (Bruineberg et al., 2018; p. 2438). At the same time, Hohwy is well aware that the skull-secluded internal model is simultaneously open to its environment. For him, the “challenge is then to balance seclusion and openness in our understanding of the mind-world relation” (Hohwy, 2016; p. 266).

For Friston himself, “the FEP [free energy principle] resolves the tension between internalist [cognitivist] and externalist [enactivist] approaches” (Allen and Friston, 2018; p. 2478). The solution takes the form of “a little bit of both”:

“Clearly, the active inference account satisfies the criteria for a radically embodied theory of mind. According to the free energy principle, an organism is best understood as a system of mutually interlocking systems; the body, mind and environment are inextricably bound up in the organism’s free energy minimization: in fact, all the heavy lifting done by active inference is in preserving a degree of (statistical) separation between the body, mind and environment” (Allen and Friston, 2018; p. 2475–2476).

Although ultimately, “the FEP offers a formal path forward for enactivism. By providing a guideline to discovery, the normative principles embedded within the approach allow enactivists to go beyond arguing about the demarcations of the organism” (Allen and Friston, 2018; p. 2478). In other words, what we get is a balanced solution with a little bit more enactivism than cognitivism. The question is whether “finding the right balance” is the best we can do; whether the tension between cognitivism and enactivism is to be resolved at all.

### Tension Redoubled

What makes free energy minimization such a compelling framework is that it is grounded in the necessary preconditions for the existence of the organism (Allen and Friston, 2018; p. 2473). If this claim is justified, and if the cognitivist and enactivist accounts follow logically from it, should we not consider whether the tension between both positions pertains to the anticipatory organism itself? Despite the “deep reciprocity between the embodied and environmental facts of the organism” (Allen and Friston, 2018; p. 2475), and the endless variations on transient extended cognitive systems (Clark, 2017), “the very existence of a system mandates the separation between the system and its external milieu” (Allen and Friston, 2018; p. 2473). As such, the tension between cognitivism and enactivism is not only maintained, but we can only get the extended, enactivist account through the cognitivist emphasis; through the imposition of a boundary. It only makes sense to speak of the organism on account of the existence of a minimal separation between organism and surroundings. Is this not the same problem we encountered in Hegel? Are we not again dealing with the tension between an abstract and a relational conception; a contradiction between independence and dependence?

We can easily reframe the tension between cognitivism and enactivism in terms of the stages Hegel distinguished. The cognitivist conception of the minimization process corresponds to the organism understood as a “system of sensibility,” which subsequently is differentiated outwards through its sensory and motor nerves. These are the moments that Hegel calls sensibility and irritability, respectively. From this perspective, the organism appears as a self-subsistent entity, where all the emphasis is placed on the internal organization of the organism undergoing change in a perpetual process of reproduction. Against this abstract designation, enactivists emphasize the necessity of the relation that the organism maintains with its outside, through which the organism engages in mutual exchange with its surroundings. And yet, the necessity of this relation depends on the existence of a self-subsistent entity. Without seclusion, there would be no open exchange, since there would be no system to engage in exchange. The paradox is that the possibility of perpetual exchange only arises from a situation in which an organism maintains a boundary between itself and its surroundings. The other way around also holds: the organism maintains its seclusion only by engaging in an open exchange with its outside. Thus, by maintaining their precarious minimal degree of autonomy, it implies that “organisms, by being organizationally closed, are also necessarily thermodynamically open” (Marques and Brito, 2014; p. 99). However, as in the case of Hegel, we are not just dealing with tension between conceptual formulations: Friston’s anticipatory organism itself sustains tension.

For the organism, there is no way to establish the accuracy of its anticipations. All the organism can do is minimize the discrepancy between its anticipations and its elicited sensory states, by either changing its anticipatory model, or its sensory states through action. In Friston’s mathematical formalizations, the minimization problem revolves around the difference between probability distributions already present on the one hand and inferred probability distributions based on new input on the other. Indeed, “this difference is always positive” (Friston, 2010; p. 128). Said differently, since minimizing free energy “places an upper bound on the entropy or dispersion of sensory states” (Friston, 2012; p. 2), the implication is that the system is continually subject to dispersion in need of bounding. The organism cannot let its sensory states disperse indefinitely if it is to maintain a minimum of consistency. At the same time, because the organism operates from within its own bounded organization, without direct access to its hidden external states, the organism also cannot get rid of dispersion entirely. Concretely, dispersion is nothing but the anticipatory error the organism sustains in the convergence on every state it takes up. As the organism moves through different states, each configuration is simultaneously marked by error; each anticipation is subject to disruption. The continuous state of tension under free energy minimization resides in the inseparable combination of anticipatory state “plus” disruption. Two questions need to be asked at this point. First, where is this tension located? Are we dealing with tension between the organism and its surroundings, or is it a tension within the organism itself? Second, how to conceive of this tension? Is its solution a goal the organism strives towards, or is it a problem concurrent with and constitutive of the organism’s existence?

### Contradiction Again

While both cognitivists and enactivists recognize the necessity of an enduring tension under free energy minimization, they differ in their conception of this tension. For enactivists, the organism maintains an “ever present dis-attunement between environmental dynamics and internal dynamics” (Bruineberg et al., 2018; p. 5). At first glance, this formulation makes perfect sense. After all, the organism needs to maintain itself in the face of external nature. We found a similar formulation in Hegel, where the organism is directed towards the outer world “as being inwardly in a state of tension towards it” (Hegel, 1830/2004; p. 381, §357). The crucial difference is that in the case of Hegel, the organism is *inwardly* in a state of tension towards its surroundings. While it is of course true that the organism stands in tension or dis-attunement toward its surroundings, external nature does not act directly on the organism’s internal organization, because this internality is shielded by a boundary comprised of sensory and action states. Since the relationship between the organism’s internal organization and its external states is mediated by this boundary, there is no tension operative directly between the organism and its surroundings; between internal and external dynamics. Instead, this tension is sustained internal to the organism’s organization; namely, between the organism’s internal anticipatory model and its sensory states. As hairsplitting as this distinction may seem, it is crucial, as we will see below.

From the perspective of cognitivism, both the unsolvable status, as well as the internality of the minimization problem is recognized: the “prediction error or free energy bound on surprise is never zero” (Hohwy, 2013; p. 172). As such, the organism “is engaged in an internal struggle to make its states fit with its input” (Hohwy, 2013; p. 179). However, from this perspective, the minimization problem becomes a “moving, ultimately unobtainable goal” (Hohwy, 2013; p. 174). The question is whether this formulation is justified. Can we say that the organism strives towards resolving its own minimization problem? What would such a solution entail? Can there be a living organism that is not subject to the imperative to minimize free energy?

With these questions, we touch on the paradoxical status of free energy minimization. There is no room in Friston’s framework for a grand conclusion where the organism succeeds definitively in the minimization of free energy. At least, not if the organism is to maintain itself as a distinct entity. From the perspective of Friston’s framework, a bounded organization necessarily engages in free energy minimization, but for minimization to take place there needs to be a bounded organization. For the living organism, this means that the only way to escape from the imperative to minimize; to escape from this state of tension, is to give up its bounded organization. In other words, death is the only definitive solution to the problem of free energy minimization. The paradox is that an unresolvable state of tension arises concurrently with the imposition of a boundary; with the precondition for the organism’s existence. It is because of the concomitance of boundary and tension that we need to invert our perspective, to the point where we repeat the procedure of Hegel’s philosophical exposition with regards to Friston’s framework; in order to enact the shift from unsolvable tension to contradiction constitutive of the organism’s life.

With this shift, we return to the opposition between external and internal purposiveness, and the problem of natural ends. In the same way that the tension between cognitivism and enactivism is not a sole theoretical tension, but simultaneously operative at the level of the organism itself; the opposition between external and internal purposiveness does not pertain only to the level of explanation. We are not just dealing with the problem of how to ground an explanation of the organism in the preconditions for the existence of that organism; the problem is equally that which grounds the organism’s perpetual anticipatory activity. In the same way that the organism in Hegel sustains contradiction between habitual structure and deficiency, Friston’s anticipatory organism sustains contradiction between internal states and dispersion. In both the case of Hegel and Friston, the role of dispersion and deficiency is that of lack positivized: an interruption which nonetheless plays a “positive” role in the internally directed revision, or externally directed activity it drives.

To take Hegel’s example of hunger, it is on account of the organism’s internal deficiency that it seeks out, ingests, and digests food. If the organism is to maintain its life, it cannot let its deficiency run amok; if it does not address its hunger, it will die. In this sense, the Hegelian organism also bounds deficiency. The great advantage of free energy minimization is that the basic logic of Hegel’s elementary example of hunger is truly “particularized in an infinite variety of ways” (Hegel, 1830/2004; p. 388, §360). With Friston’s framework, the basic logic of Hegel’s dialectical process is generalized to every aspect of the organism’s perceptual and motor capacities, whereby the entirety of the organism’s internal structure “feeds off” its surroundings. If Hegel’s contradictory organism is a process that revolves around its own deficiency, then free energy minimization is a process which subsumes and bounds all of the organism’s deficiencies. Not only do both free energy minimization and activity of deficiency pertain to a perpetual process of self-organization and self-production, but free energy minimization is activity of deficiency.

Due to the recurrence of deficiency, and the concomitance of this recurring problem with the existence of a boundary, it is insufficient to formulate the minimization of free energy in terms of an unobtainable goal. For the organism, there is no unobtainable goal, because the “goal” consists in sustaining the continuous process itself, through which dispersion or lack inherent to the organism’s organization is constrained, and a boundary is maintained. We could say the “goal” is continually “reached” and “missed” simultaneously. A definitive solution to the problem of free energy minimization is repeatedly missed, but this miss is the goal itself. More precisely, we are dealing with the “splitting between goal and aim, the moment when the true aim is no longer to hit the goal but to maintain the very circular movement of repeatedly missing it” (Žižek, 1993; p. 199). In this sense, the organism exists as a natural end: the process of free energy minimization is without end because the end is the process of free energy minimization itself. As such, the direction of boundary-maintenance is not outwards: in the attempt of biological systems to maintain their states and form, we are dealing with a process that is thoroughly self-directed.

In addition to the designation of the organism’s self-movement in terms of self-organization, self-production, and self-determination, perhaps it is self-limitation which best captures the convergence between Hegel and Friston, and simultaneously announces their minimal, but crucial difference. As the organism revisits a limited number of states that lie within its physiological bounds, it engages in a limiting movement of the “self” directed at the “self”: “life emerges when external limitation (of an entity by its environs) turns into self-limitation” (Žižek, 2006; p. 205). This is the process that is central both in the case of Hegel and Friston: everything hinges on the existence and maintenance of a boundary. No boundary, no organism.

At the same time, self-limitation is operative in terms of a limitation (lack) at the level of the “self”:

“Where there is a limitation [*Schranke*], it is a negation only for a third, for an external comparison. But it is lack only in so far as the lack’s overcoming is equally present in the same thing, and contradiction is, as such, immanent and explicitly present in that thing” (Hegel, 1830/2004; p. 385, §359).

While both Hegel and Friston emphasize the necessity of an organization that is bounded [*beschränkt*], only in Hegel we find the primacy of contradiction constitutive of the process that sustains such bounded organization. If we enact the shift from tension to contradiction, the unsolvable status of the minimization problem functions not as an unobtainable goal but serves first and foremost as the condition both for the organism’s existence as well as its perpetual free energy minimization. Only in trying to overcome the unsolvable obstacle of its persistent deficiency or dispersion does the organism continue to anticipate: it is this continually present inherent limitation around which the organism’s anticipatory activity circulates. For this reason, the tension between organism and surroundings needs to be transposed back into the organism, to the point where we conceive of this tension as the organism’s constitutive contradiction.

To return to the opening problem of this section: the tension between internalist and externalist approaches is not only retained; it is elevated to a contradiction constitutive of the perpetual process of free energy minimization. If the organism’s founding gesture is the imposition of a boundary, then this gesture simultaneously condemns the living being to a life of sustained contradiction. This is the fundamental dialectic of Friston’s free energy minimization expressed in the opposing positions of cognitivism and enactivism.

**Figure 1:**
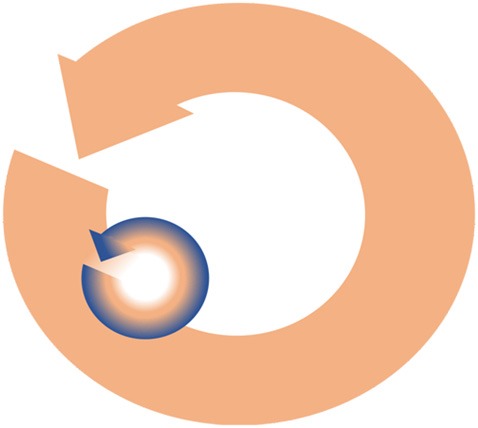
Schematic representation of the contradictory organism. Every organism (blue) operates within the dynamics of its surroundings (brown). The organism’s structure as distinct from its surroundings is maintained only as a continuous process of self-organization and self-production, revolving around recurring lack or dispersion (white) internal to the organism’s organization (see Friston, 2005; Figure 1). The organism’s internal habitual model is a representation of, and “feeds off,” its surroundings.

This perspective dissolves the so-called “dark-room problem”: the question concerning why, if an organism is primarily concerned with the minimization of prediction error/free energy, it does not simply seek out a dark and silent place: if there is no input to the system, then there is nothing to minimize. This is a problem only if we conceive of the organism as a fully autonomous and constituted entity, which subsequently engages in minimization. From a Hegelian perspective, it is a problem only if we remain at the level of an abstract designation in terms of sensibility and irritability. If instead, we follow Hegel to the very end, the organism’s constitutive contradiction appears equally necessary for the organism’s existence; no less so than the existence of a boundary. From this perspective, the organism’s activity is grounded not in some positive aspect of its organization, but in the sustained negation of this organization, around which the organism’s anticipatory self-production circulates (see Figure 1). In dealing with the dark-room problem, it is not enough to emphasize the need for continued scientific work; how, in “due course, realistic working models will be forthcoming, at which stage this philosophical debate will rightly give way to detailed empirical evaluation of the claims being made” (Friston et al., 2012b; p. 6). While Friston’s declaration and willingness to abandon his framework in the face of failure is admirable (a stance admittedly missing in Hegel, see Žižek, 2012; p. 462); perhaps there will not be a need to “search for a better model!” (Friston et al., 2012b; p. 6). Hegel’s philosophy is well able to dispose of “philosophical debates” such as the dark-room problem on its own. And if we enact the Hegelian shift from tension to contradiction with regards to free energy minimization, so is Friston’s framework.

## Discussion

This article sprang from the convergence between Hegelian dialectics and Friston’s approach to the brain. We set out to answer two questions. First, is free energy minimization a formalization of the dialectical process of plasticity, as understood by Malabou? We saw that indeed, in the coincidence of free energy minimization and the Hegelian notion of plasticity, Friston provides us with an approach to the brain which reinvigorates Hegelian dialectics from the perspective of neuroscience. In both cases, we are dealing with a perpetual process in which form is received and produced, and where *formation* and *deformation* occur simultaneously. Whether we speak in terms of a habitual anticipatory structure, or a model comprised of anticipatory states, what is important is that in both cases, the organism does not *have* a model, but *is* a model; there is no organism outside the process wherein its anticipatory structure is continually reproduced and transformed.

The second question central to this article was this: can we enact the Hegelian shift from tension to contradiction with regards to free energy minimization? This question touched on two tensions simultaneously: theoretical and actual. In Hegel’s work, we saw that the determination of the organism took the form of a succession of stages, passing from abstract to relational; from independence to dependence. However, the tension between these designations was not resolved. On the contrary, this tension needed to be located and mobilized in the actual workings of the organism itself. This is where we encountered the shift from tension to contradiction. If tension pertains to an opposition *between* concepts (e.g., seclusion and openness) or entities (e.g., organism and surroundings), then contradiction is an antagonism internal to one of the terms. In our case, we saw that this contradiction pertains to the organism’s simultaneous maintenance of identity, as well as the negation of this identity in the form of recurring lack or deficiency. The attempt to answer the second question central to this article could be viewed as nothing more than the attempt to repeat Hegel’s procedure with regards to Friston’s free energy minimization. First, we regarded the conceptual tension between cognitivism and enactivism; between the emphasis on seclusion and openness. We tried to cast this tension as the succession from an abstract to a relational conception of the organism. Second, instead of trying to resolve this tension, we shifted our attention to the organism’s actual tension under free energy minimization; namely, the organism’s unrelenting imperative to minimize entropy, dispersion, or anticipatory “error.” In this sense, both “lack” in Hegel, and “dispersion” in Friston, serve the same structural role: they threaten the perpetuation of the organism’s organization and as such, must be kept within bounds. Finally, because the organism’s contradiction between identity and negation exists concurrently with the existence of a boundary, it is constitutive for the organism’s existence and at the basis of the organism’s perpetual anticipatory activity. As such, the shift from tension to contradiction allows us to see how Friston’s free energy minimization is a modern instantiation of Hegelian dialectics.

While we appealed to Hegel in order to bring to light the contradictory tension at the center of free energy minimization, we should emphasize that Friston’s framework goes beyond Hegel in the generalization of the rudimentary example of hunger to the organism’s functioning in the broadest possible sense, to the point where the logic of plasticity pervades every aspect of the organism’s functioning. Furthermore, Friston’s framework goes beyond Hegel in the specification of the concurrent processes of anticipation and precision estimation. Not only is the organism an anticipatory structure, but the transience of this structure depends on the continuous interplay between the anticipatory state, the precision of this state, as well as the precision afforded to the disruption of this state. As such, free energy minimization formalizes the mechanism that determines an organization’s plasticity, in which the Hegelian legacy is maintained.

## Author Contributions

EB wrote the initial draft of the manuscript. Both EB and HS made significant changes to the initial draft in order to arrive at the final version.

## Conflict of Interest Statement

The authors declare that the research was conducted in the absence of any commercial or financial relationships that could be construed as a potential conflict of interest.

## Footnotes

^1^There is much debate about what contradiction means in Hegel’s philosophy (e.g., see Bole, 1987; Bordignon, 2017). While we may define contradiction as a pair of sentences, where one negates the other, it is important to emphasize that we are concerned not solely with epistemological contradiction. In Hegel’s words, this means letting go of “a tenderness for the things of this world” (Hegel, 1830/2004; p. 5, §245), in order to assert the stain of contradiction at the level of things themselves. Over the course of this article, we follow Žižek in the search for “‘contradiction’ [inconsistency, impossibility, antagonism] as an internal condition of every identity” (Žižek, 1989; p. xxix). In our case, this search pertains to the living organism under free energy minimization.

^2^In classical logic, the principle of explosion is often expressed as *ex contradictione quodlibet sequitur* (from contradiction, anything follows; e.g., see López-Astorga, 2016). In Malabou’s work, “explosion” pertains to plasticity as “the capacity to annihilate the very form it is able to receive or create.” For example, the French “*plastique*, […], is an explosive substance” (Malabou, 2004/2008; p. 5). In this sense, while plasticity enables a structure of anticipation, it simultaneously upholds the possibility of this structure to deform itself, as well as radical openness towards the future.
